# Glycosides from Marine Sponges (Porifera, Demospongiae): Structures, Taxonomical Distribution, Biological Activities and Biological Roles

**DOI:** 10.3390/md10081671

**Published:** 2012-08-10

**Authors:** Vladimir I. Kalinin, Natalia V. Ivanchina, Vladimir B. Krasokhin, Tatyana N. Makarieva, Valentin A. Stonik

**Affiliations:** G.B. Elyakov Pacific Institute of Bioorganic Chemistry, Far-Eastern Branch of the Russian Academy of Science, Vladivostok, Prospect 100 letya Vladivostoka, 159, Russia; Email: kalininv@piboc.dvo.ru (V.I.K.); ivanchina@piboc.dvo.ru (N.V.I.); kras@piboc.dvo.ru (V.B.K.); makarieva@piboc.dvo.ru (T.N.M.)

**Keywords:** glycosides, sponges, structures, activities, taxonomic distribution, biological functions

## Abstract

Literature data about glycosides from sponges (Porifera, Demospongiae) are reviewed. Structural diversity, biological activities, taxonomic distribution and biological functions of these natural products are discussed.

## 1. Introduction

Triterpene and steroid glycosides are known as widespread secondary metabolites in terrestrial higher plants. Although they are comparatively rare chemical constituents in the animal kingdom, numerous marine “animal” triterpene glycosides were also isolated from sea cucumbers (Holothurioidea, Echinodermata), in which they have been found in all the orders of this class of the phylum Echinodermata and possess a strict taxonomical specificity [1,2,3,4,5,6]. Steroid glycosides from starfish (Asteroidea, Echinodermata) compose another well-known group of bioactive marine glycosides, attracting much attention from chemists and pharmacologists for a long time [7,8,9]. Marine sponge glycosides were discovered later in comparison with those from echinoderms, but compounds of this chemical class were also shown to be a large and diverse class of bioactive amphiphilic natural products. Recently, some of them were partly reviewed along with different bioactive sesterterpenes and triterpenes isolated from sponges [10]. In the present review, we attempt to cover literature data, concerning main directions in the studies on sponge glycosides, including structural diversity, physiological activities, taxonomic distribution and biological roles. Herein, we have processed and discussed the data about four subclasses of these substances: (i) tetracyclic triterpene glycosides; (ii) other triterpene glycosides; (iii) steroid glycosides, and (iv) glycosides of non-isoprenoid aglycones, but not of such specific groups as cerebrosides, nucleosides and tetramic acid glycosides from sponges.

## 2. Tetracyclic Triterpene Glycosides

### 2.1. The Order Astrophorida

*Melophlus sarasinorum* (family Ancorinidae) is a sponge widely distributed in the Indo-West Pacific region including Great Barrier Reef, Indonesia, Palau Islands, *etc*. It is also known in literature as *Asteropus sarasinorum*, *Melophlus isis* and *Stellettinopsis isis* as junior synonymic names of the same species [11]. Moreover, erroneous names of this species were used in chemical literature such as *Asteropus sarasinosum* [12,13] and *Melophlus sarassinorum* [14]. Herein we use the valid taxonomical name.

The glycoside fraction of *Melophlus sarasinorum* contains complicated mixture of 14-nor-methyl-lanostane (or 30-norlanostane) triterpene glycosides. First, nine glycosides, sarasinosides A_1_ (**1**), A_2_ (**2**), A_3_ (**3**), B_1_ (**5**), B_2_ (**7**), B_3_ (**9**), C_1_ (**4**), C_2_ (**6**), and C_3_ (**8**), were isolated and structurally elucidated by Kitagawa *et al.* [12,15] from the Palauan sponge. Sarasinoside A_1_ (**1**) was independently and simultaneously isolated by Schmitz *et al.* [13] from the sponge collected near Guam Island and from another, Truk Lagoon collection. Other representatives of the same structural group, namely sarasinosides D (**10**), E (**11**), F (**12**), G (**13**) and H (**14**) along with known sarasinoside B_1_ (**5**), were isolated by Espada *et al.* from the Guam Island population of the same species [16].

Lee *et al.* have isolated four new 14-nor*-*methyl-lanostane glycosides, sarasinosides H_1_ (**14**), H_2_ (**15**), I_1_ (**16**) and I_2_ (**17**) along with known sarasinosides A_1_ (**1**) and A_3_ (**3**) from the sponge, collected off Guam [17]. Dai *et al.* have isolated new 14-nor-methyl-lanostane glycosides, sarasinosides J (**18**), K (**19**), L (**20**) and M (**21**) along with known sarasinosides A_1_ (**1**), A_3_ (**3**), I_1_ (**16**), I_2_ (**17**) and H_2_ (**15**) from *M. sarasinorum* collected from Sulawesi, Indonesia [14]. Finally, Santalova *et al*. [18] have isolated two new 14-nor-methyl-lanostane triterpene glycosides, sarasinosides A_4_ (**22**) and A_5_ (**23**), along with known sarasinosides A_1_ (**1**), A_2_ (**2**), A_3_ (**3**), M (**21**) and L (**20**) from the Australian collection of the same species.

**Chart 1:**
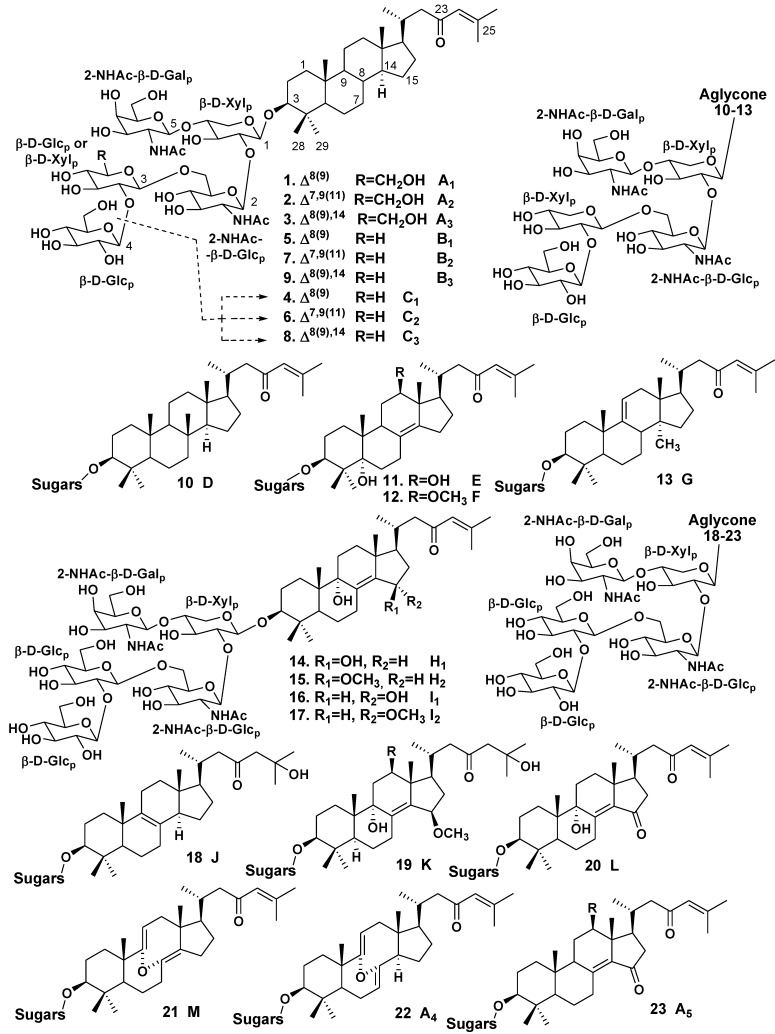
Structures of sarasinosides.

Most aglycones of sarasinosides have the norlanostane skeleton system with 8(9)-, 9(11)- or 8(14)-double bonds in tetracyclic moieties and identical 23-keto-Δ^24(25)^ side chains, although glycosides **2**, **6** and **7** have 7(8),9(11)-diene system, while the glycosides **3**, **8** and **9** possess 8(9),14-diene system. The most unusual aglycone moiety of sarasinoside D (**10**) contains a methyl group at C-8 instead of the usual C-14 position. The rearrangements led to relative compounds in certain triterpenoids isolated from higher plants is carried out via intermediates with Δ^14(15)^-unsaturation [16]. More oxidized aglycones in sarasinosides E, F, H_1_, H_2_, I_1_, I_2_, J–L and A_5_ (**11**, **12**, **14**–**20**, **23**, respectively) contain additional oxy-, methoxy- or ketone groups, while aglycones of sarasinosides M and A_4_ (**21**, **22**) are 8,9-*seco*-derivatives having unique 8α,9α-epoxy-8(14),9(11),24-triene and 8α,9α-epoxy-7(8),9(11)-diene structural fragments (Chart 1) [14,15,16,17,18].

Carbohydrate chains of all the glycosides are very similar to each other and have the same architecture. All sugar units (xylose, two glucoses, 2-(*N*-acetylamino)-2-deoxy-galactose and 2-(*N*-acetylamino)-2-deoxy-glucose) belong to D-series and are in pyranose forms, the configuration of all the glycosidic centers is β. Glycosides **1**, **2** and **3** and many other glycosides of this structural series are pentaosides and have D-glucose as the third monosaccharide residue, while glycosides **4**, **6** and **8** are tetraosides containing identical carbohydrate chains with xylose residue as the third and the terminal sugar unit.

Thus, totally 23 sarasinosides were isolated from *Melophlus sarasinorum*. Most of these glycosides are pentaosides and their carbohydrate chains may differ in the third monosaccharide unit (glucose or xylose) only. Structural diversity of sarasinosides is mainly provided both by oxidative processes in rings B, C and D and different positions of double bonds in the tetracyclic part of aglycones.

Data, concerning biological activities of sarasinosides are limited. Kitagawa *et al.* have compared ichthyotoxic action of sarasinosides A_1_ (**1**) and B_1_ (**5**) against killifish *Poecilia reticulata* [12].Glycoside **1** with glucose as the third monosaccharide unit showed LD_50_ of 0.39 μg/mL, while glycoside **5 **with xylose in this position demonstrated LD_50_ of 0.71 μg/mL. Glycosides **1** and **5** have the same moderate inhibitory activities (ED_50_ of 10 μg/mL) against fertilized eggs of starfish *Asterina* (=*Patiria*) *pectinifera*. Schmitz *et al.* reported the cytotoxicity of sarasinoside A_1_ (**1**) against human lymphocytic leukemia cell line (ED_50_ of 2.8 μg/mL) [13]. Lee *et al.* indicated cytotoxic activities of sarasinosides A_2_ (**2**) and A_3_ (**3**) against human leukemia cell line K562 (ED_50_ of 6.5 and 17.1 μg/mL, respectively), while sarasinosides H_1_ (**14**), H_2_ (**15**), I_1_ (**16**) and I_2_ (**17**) were not active in the test [17]. Dai *et al.* showed that sarasinoside A_1_ (**1**) possesses strong activity against the yeast *Saccharomyces cerevisiae*, but it is inactive against the bacteria *Bacillus subtilis* and *Escherichia coli*. Sarasinoside J (**18**) was also strongly active against *S. cerevisiae*, but possessed the moderate activity against *B. subtilis* [14]. Hence, we may conclude that sarasinosides with common 8(9)-double bond or 7(8),9(11)-diene system possess strong or moderate cytotoxic activities against tumor cell lines, yeast, fertilized eggs of starfish and ichthyotoxicity. Modifications in C and D rings, including the migration of double bond into 8(14)-position and introduction of oxygen-bearing substituents as well as other oxidative transformations in cyclic systems of aglycones decrease the activities.

*Erylus formosus* (the family Geodiidae) is widely distributed in shallow waters of the Caribbean Sea and the Eastern Brazil and the most studied sponge in relation of its tetracyclic triterpene glycosides. Jaspars and Crews have isolated lanostane tetraoside formoside (**24**) from extracts of the sponge arose during an expedition to the Bahamas [19]. The aglycone of formoside belongs to the lanostane derivatives, containing 8(9)- and 24(25)-double bonds and a carboxy group at C-14. It was earlier found from *Penares* sp. as a free triterpene and named penasterol [20], while its 3-*O*-acetyl- and 3-oxo-derivatives were also isolated from the sponge *Penares incrustans* [21]. All sugars (two galactoses and two arabinoses) are in pyranose forms.

As a result of studies on *E. formosus*, collected off the Bahamas by scuba at the depth of 55 feet, Stead *et al.* have isolated the related lanostane bioside eryloside F (**25**) with the same aglycone, also containing arabinose as the first sugar attached at C-3 of the aglycone and the second sugar unit (galactose) linked at C-2 of the arabinose residue [22].

Another glycoside of this series, formoside B (**26**) was found by Kubanek *et al.* in the sponge collected also off the Bahamas along with the known formoside (**24**). Glycoside **26** differs from **24** in the presence of 2-(*N*-acetylamino)-2-deoxy-D-galactose instead of a terminal galactose. The same group of scientists isolated also a series of fractions, containing related hexaosides and triosides and characterized these fractions by mass-spectrometry. However, individual glycoside components were not obtained [23].

A difficult task of separation of similar fractions was successfully solved by Antonov *et al.*, who isolated a series of individual lanostane biosides, triosides and hexaosides [24,25] from the Mexican collection of the sponge. In these glycosides, all sugars are in pyranose forms, belong to D-series and have β-configuration of glycosidic bonds, except arabinose residues, which belong to L-series and have α-configuration of glycosidic bonds. Erylosides F_1_–F_4_ (**27**–**30**) were proved to be close related analogs of eryloside F (**25**) differing from each other in the aglycone side chains [23]. Previously known eryloside F (**25**) has also been isolated. Glycoside **27** contains the aglycone similar to penasterol with the side chain, having a methylene at C-24. Penasterol and these aglycones are predominant non-carbohydrate moieties in the glycosides from *Erylus* spp. Glycoside **28** has a 25(26)-double bond and a 24*R*-hydroxy group, while glycoside **29** contains 25(26)-double bond and 24*S*-hydroxy group. Eryloside F_4_ (**30**) is a 24-keto-derivative of glycosides **28** and **29**. Two new triosides, erylosides M (**31**) and N (**32**), were also isolated by this group from the same species along with eryloside H (**33**), earlier known from *Erylus nobilis* [26]. Glycoside **32** has penasterol as aglycone, while **31** contains aglycone with a methylene group at C-24 in the side chain. The previously known glycoside **33** differs from eryloside M only in the presence of 2-(*N*-acetylamino)-2-deoxy-D-galactose instead of a galactose residue. A new tetraoside eryloside O (**34**) has a similar structure to that of formoside (**24**), but the third terminal monosaccharide residue was identified as α-L-arabinose instead of D-galactose in formoside. New hexaosides, erylosides P (**35**) and Q (**36**), have the same carbohydrate chains and differed in the side chains of aglycones. The carbohydrate moieties of both glycosides are similar to that of formoside (**24**), but have an additional glucose residue attached at C-4 of the fourth sugar (galactose) and a terminal xylose residue, attached at C-2 of this glucose residue [24].

In continuation, Antonov *et al.* have isolated one more trioside eryloside R_1_ (**37**) along with the known formoside (**24**), and six new hexaosides erylosides T_1_–T_6_ (**38**–**43**) [25]. All their sugars are also in pyranose forms, belong to D-series, and have β-configurations for the glycosidic bonds, except L-arabinose residues. Glycoside **37** has penasterol as aglycone, its terminal galactose is linked to C-3 of the first monosaccharide residue (arabinose), while another galactose unit is attached to C-2 of the arabinose. Hexaosides **38**-**40** also contain penasterol as the aglycone, while hexaosides **41**–**43** contain another distributed in this series aglycone with a methylene group at C-24 in side chains. All the hexaosides possess carbohydrate chains having the same general structural plan and their carbohydrate chains are similar to those of erylosides P (**35**) and Q (**36**).

It should be noted that not only hexaosides, but all the triterpene glycosides, isolated from *Erylus formosus* (totally 20 compounds) possess similar structural features. All of them contain the same α-L-arabinopyranose as the first monosaccharide residue, attached to C-3 in the aglycone moieties. The number of sugars may be two, three, four or six in these compounds.

**Chart 2:**
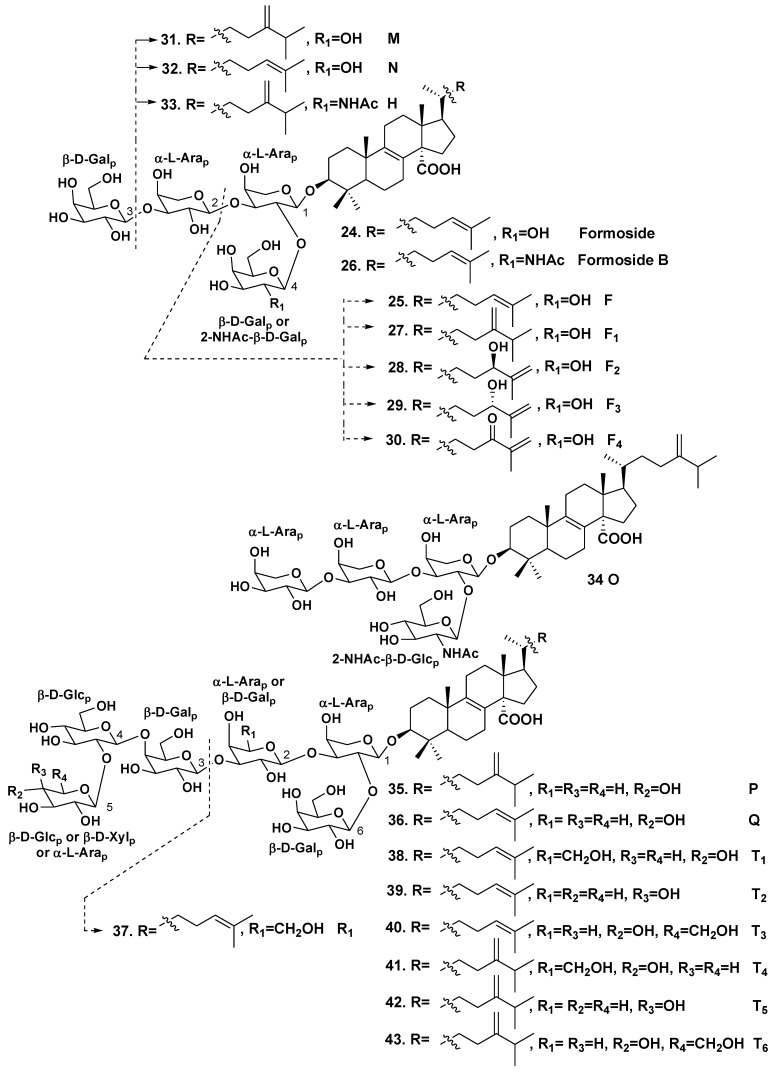
Erylosides from *Erylus formosus*.

One of terminal sugars is linked to C-2 of the first L-arabinose residue. The hexaosides contain also another terminal monosaccharide unit, attached to C-2 of the fourth sugar. All other linkages between monosaccharides are 1,3, except a linkage between the third fourth and the fourth monosaccharide units, which is 1,4. Not only arabinose, glucose and galactose, but also 2-(*N*-acetylamino)-2-deoxy-D-galactose were found as terminal monosaccharide units in the glycosides of *Erylus formosus* (Chart 2).

Probably, the biosynthesis of carbohydrate chains is initiated in this species by a glycosyltransferase bearing L-arabinosyl residue. Elongation of carbohydrate chains by other glycosyltransferases results in possibility of monosaccharides to be replaced by others in different triterpene glycosides of *E. formosus* retaining the general structural plan of carbohydrate chains [25].

Eryloside F (**25**) was proved to be a potent thrombin receptor antagonist, inhibiting human platelet aggregation *in vitro*. This glycoside facilitates the calcium mobilization in cells registered by FLIPR assay [22]. Formoside (**24**) shows antiviral activity (IC_50_ of 3.5 μg/mL *vs.* HSV-1) and modest antibacterial action (IC_50_ of 31.3 μg/mL *vs. Corynebacterium xerosis*) [19]. The fractions of hexaosides were active against amphotericin B-resistant *Candida albicans* (IC_50_ of 3.9 μg/mL) [23]. Eryloside F_3_ (**29**), but not its epimer eryloside F_2_ (**28**) induces the early apoptosis of Ehrlich carcinoma cells at the concentration of 100 μg/mL, while eryloside F_1_ (**27**) and eryloside F (**25**) activate the Ca^2+^ influx into mouse spleenocytes (130% of the control) at the same dose [24].

*Erylus lendenfeldi* is a species distributed in the IndoPacific geographical area. A new bis-nor-triterpene bioside eryloside A (**44**), containing 4β,14-di-nor-methyl-lanostane aglycone and β-D-galactopyranose residue as the first sugar residue together with another (terminal) β-D-galactopyranose residue attached to C-2 of the first monosaccharide residue in carbohydrate moiety, was isolated from the Red Sea specimen of the sponge by Carmely *et al*. in 1989 [27]. The aglycone of **44** contains 8(9)- and 14(15)-double bonds and a hydroxy group at C-23.

**Chart 3:**
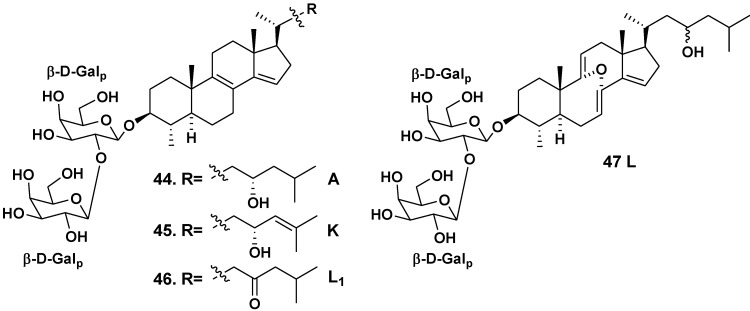
Erylosides from *Erylus lendenfeldi*.

Later Sandler *et al*. [28] from Faulkner’s laboratory found the same glycoside in another the Red Sea collection of the sponge. In addition to **44**, two new related compounds were obtained and named as erylosides K and L (**45**,**46**). The absolute configuration of the C-23 asymmetric center in **44 **and**45** was established through the corresponding MTPA-esters with previous hydrogenation of **45** using rhodium as a catalyst. The containing 24(25)-double bond in its aglycone eryloside K (**45**) was independently and almost simultaneously isolated by Fouad *et al*. [29] at the studies on the same sponge, collected off the Jordan coast in the Gulf of Aqaba, the Red Sea. Eryloside L (**46**) was structurally identified by Sandler *et al*. as the corresponding 23-ketone [28]. Fouad *et al*. isolated also similar glycoside **47**, having a rare 8α,9α-epoxy-8,9-seco-7,9(11),14-triene fragment in the aglycone part (Chart 3) [29].

Bioside **47** was also named as eryloside L. Therefore, two different glycosides (**46** and **47**) are known now under the name “eryloside L”. We propose rename the compound **46** to eryloside L_1_, because **46** was later reported.

Glycoside **44** shows cytotoxic activity against P388 tumor cell line (IC_50_ of 4.2 μg/mL) and antifungal activity against *Candida albicans* (MIC of 15.6 μg/mL) [27]. This glycoside was also active against *Escherichia coli*, *Bacillus subtilis* and *C. albicans* (zones of inhibition at 10 μg per disc were of 6, 7 and 7 mm, respectively). Glycosides **44** and **45** lead to mortality rate of 50% at a concentration of 0.1 μg/mL in the brine shrimp assay, while compound **46** was inactive in the test. Glycosides **44**, **45**, and **47** did not show cytotoxicity against JURKAT, THP-1 and MM-1 tumor cell lines [29]. Glycosides **44**–**46** were selectively active against Δ*rad50* budding yeast strain (0.8, 2.0 and 3.4 μg/mL) in comparison with activity against wild parent yeast strain (IC_50_ of 3.5, 6.1 and 11.4 μg/mL, correspondingly). It is known that selective cytotoxicity against similar mutant yeast may be an indicator of topoisomerase poisons. However, the activities against TOP1oe (IC_50_ of 5.7, 7.5 and 10.9 μg/mL) and TOP2oe (10.8, 12.2 and 9.5 μg/mL) were milder than those of the known topoisomerase inhibitors such as camtothecin and idarubacin. The aglycone, obtained from glycoside **44**, was inactive against the yeast strain that indicated a significant role of carbohydrate chain in the activity [28].

**Chart 4:**
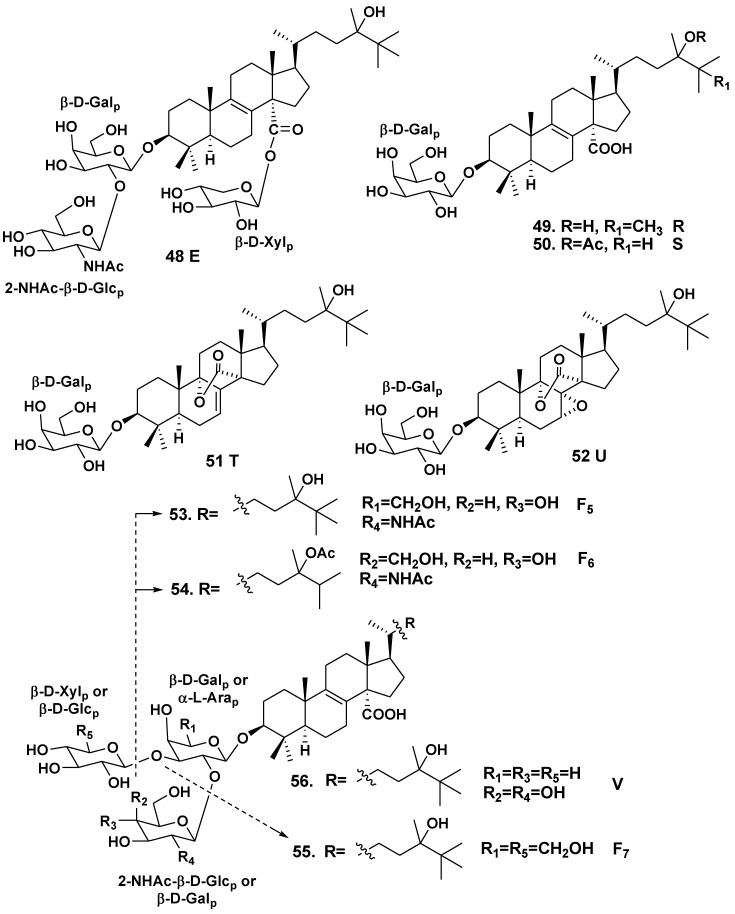
Erylosides from *Erylus goffrilleri*.

*Erylus goffrilleri* is an Atlantic tropical sponge species. Gulavita *et al*. isolated unusual lanostane glycoside, eryloside E (**48**), having not only a bioside carbohydrate chain consisted of galactose as the first sugar and 2-(*N*-acetylamino)-2-deoxy-D-glucose attached to C-2 of the galactose, but also another carbohydrate fragment composed of xylose, linked to a carboxy group at C-14 of the aglycone [30]. The aglycone has 8(9)-double bond, hydroxy-group at C-24 and additional methyl groups at C-24 and C-25 (Chart 4).

Afiyatullov *et al.* have isolated four related lanostane monosides, erylosides R (**49**), S (**50**), T (**51**) and U (**52**) with β-D-galactopyranose as a carbohydrate moiety [31]. Glycoside **49** contains the lanostane aglycone with 8(9)-double bond and carboxy group at C-14, hydroxy group at C-24 and two additional methyl groups at C-24 and C-25 in the side chain. Glycoside **50** contains a very similar aglycone with acetate and an additional methyl groups at C-24 in the side chain. The aglycone of glycoside **51** featured the same side chain as in **49**, but has 7(8)-double bond and an uncommon lactone function between С-14 and С-9. Glycoside **52** is very similar to **51**, but contains an additional 7α,8α-epoxy group.

In addition, Afiyatullov *et al.* have also isolated three lanostane biosides, erylosides F_5_ (**53**), F_6_ (**54**) and F_7_ (**55**) and one trioside, eryloside V (**56**). Glycosides **53** and **54** contain closely related carbohydrate chains consisting of D-galactose and 2-(*N*-acetylamino)-2-deoxy-D-glucose, attached to C-2 of the galactose residue [31]. The sugars are in pyranose forms and have a β-configuration of the glycoside bonds. As it is characteristic of many erylosides, their aglycones have 8(9)-double bond and a carboxy group at C-14. The side chain of the aglycone in glycoside **53** contains a hydroxy group at C-24 and two additional methyl groups at C-24 and C-25. The side chain of **54** includes an OAc-group at C-24 and only one additional methyl group at C-24. Glycoside **55** has not only the same aglycone as in **53**, but also the second sugar residue, β-D-glucopyranose, attached at C-3 of the first monosaccharide residue. Eryloside V (**56**) is a trioside with the same aglycon as **53 **and α-L-arabinose as the first sugar. Additional alkylation in side chain is a particular structural feature of erylosides from this sponge species.

Eryloside E (**48**) weakly inhibited the binding of ^125^[I]-Bottom Hunter labeled C5a to its receptor (IC_50_ > 10 μM). Eryloside E shows also an immunosuppressive activity (EC_50_ of 1.3 μg/mL), its immunosuppressive effect was specific and not caused by general cytotoxicity [30]. Erylosides R (**49**), S (**50**), T (**51**), V (**56**), F_6_ (**54**) and F_7_ (**55**) exhibit cytotoxic activities against Ehrlich carcinoma tumor cells (IC_50_ of 20–40 μM) [30], while glycosides **52**, **53** and **56** are inactive.

**Chart 5:**
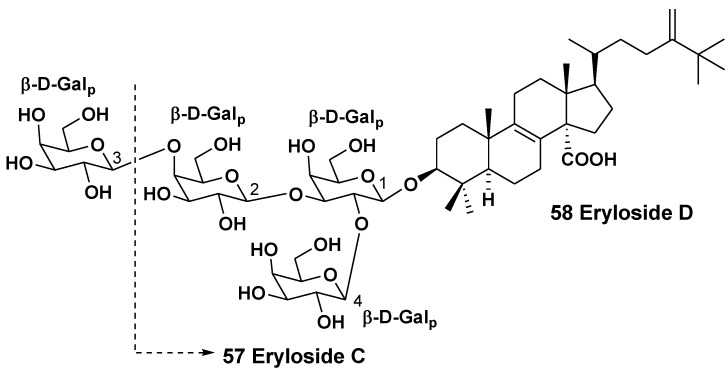
Erylosides from *Erylus* sp.

The studies on the sponge *Erylus* sp., isolated from a New Caledonian specimen, collected at a depth of 500 m yielded two lanostane saponins, trioside and tetraoside, named erylosides C (**57**) and D (**58**), correspondingly [32]. The aglycones of both glycosides possess 8(9)-double bond, a carboxy group at C-14, methylene group at C-24 and an additional methyl group at C-25. All the monosaccharide residues are β-D-galactopyranoses. One terminal sugar is attached to C-2 of the first monosaccharide residue, while another terminal sugar is linked to C-3 of the first sugar in the glycoside **57** as it is characteristic of many triterpene glycosides from sponges, belonging to the genus *Erylus*. However, one of the terminal sugars in tetraoside **58** is attached to C-4 of the second sugar, but not to C-3 (Chart 5).

**Chart 6:**
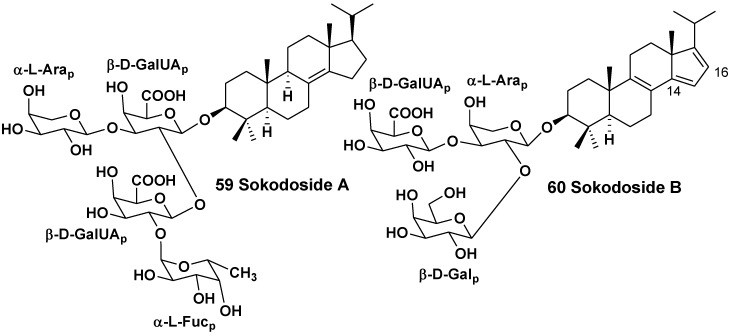
Sokodosides from *Erylus placenta*.

The related sponge *Erylus placenta* was collected in the Pacific Ocean near the shore of the South Japan (Hachijo Island). Okada *et al*. isolated two unprecedented 14-nor-methyl-23,24,25,26,27-pentanorlanostane glycosides from the sponge. A unique aglycone of sokodoside A (**59**) has 8(14)-double bond, while the closely related sokodoside B (**60**) possesses a unprecedented conjugated 8(9),14(15),16(17)-triene system [33] (Chart 6). Glycoside **59** is a branched tetraoside. The first sugar of **59** is β-D-galactouronic acid, the second monosaccharide residue attached to C-2 of the first sugar is another residue of β-D-galactouronic acid, the third (terminal) sugar attached to C-2 of the second sugar is α-L-fucose and another terminal sugar is α-L-arabinose. Sokodoside B (**60**) is a branched trioside with carbohydrate chain, consisting of α-L-arabinose, β-D-galactose and β-D-galactouronic acid. In the original paper the configuration of arabinose residues was erroneously described as β, while the coupling constant in the ^1^H NMR spectra of **59** and **60** definitely showed on a α-configuration for these L-arabinose residues.

Recently, a convergent synthesis of the trisaccharide moiety of sokodoside B was carried out through thioglycoside activation using sulfuric acid immobilized on silica in conjunction with *N*-iodosuccinimide [34]. Sokodosides A and B show moderate growth-inhibitory activities against the fungus *Mortierella ramanniana* and different strains of the yeast *Saccharomyces cerevisiae* with or without mutations (cdC_2_8, act1-1 and erg6). The size of inhibition zones ranged between 8 and 16 mm at 50 μg of the tested substance on 6 mm spot on thin paper disk. Compound **59** was more active than **60**. Glycosides **59** and **60** show moderate cytotoxic activities against P388 cells with IC_50_ of 100 and 50 μg/mL, correspondingly. Hence, there is a correlation between their antifungal and cytotoxic activities.

**Chart 7:**
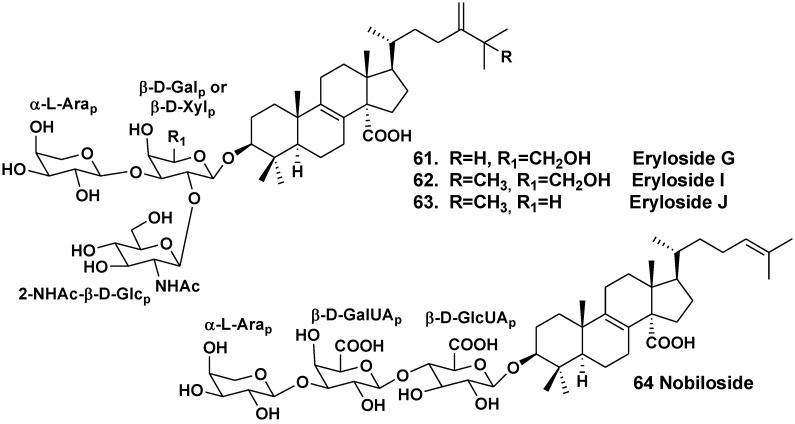
Erylosides from *Erylus nobilis*.

Shin *et al*. have isolated four lanostane triosides erylosides G (**61**), H (**33**), I (**62**) and J (**63**) from the sponge *Erylus nobilis*, collected near the South shore of Jaeju Island (South Korea, Korean Strait) [26]. The aglycones of all these glycosides have 8(9)-double bond, carboxyl group at C-14 and 24-methylene group in side chains (Chart 7). Compounds **62** and **63** have also an additional methyl at C-25, like glycosides from *E. goffrilleri*. Both glycosides are triosides. The glycosides **61**, **33**, **62** and **63** demonstrate moderate cytotoxic activities against the human leukemia cell line K562 with IC_50_ of 22.1, 24.8, 17.9 and 21.8 μg/mL, respectively.

Takada *et al*. have isolated nobiloside (**64**), a linear lanostane trioside from *E. nobilis* collected in the Pacific Ocean near Shikine-jima Island, 200 km southward Tokyo, Japan [35]. The aglycone of nobiloside contains 8(9)- and 24(25)-double bonds and carboxy group at C-14. The first sugar in the carbohydrate chain is β-D-glucuronic acid, the second sugar is β-D-galactouronic acid, a terminal sugar is α-L-arabinose. Synthesis of two trisaccharides, related to the saponins from *E. nobilis* was reported from commercially available D-galactose, L-arabinose and D-glucosamine hydrochloride via rational protecting group manipulations [36]. The glycoside shows inhibitory activity against neuraminidase from the bacterium *Clostridium perfringens* with IC_50_ of 0.46 μg/mL.

Thus, all the studied *Erylus* species contain bioactive triterpene glycosides, in majority having 14-carboxy lanostane aglycones and carbohydrate moieties with predominance of arabinose and galactose.

### 2.2. The Order Poecilosclerida

**Chart 8:**
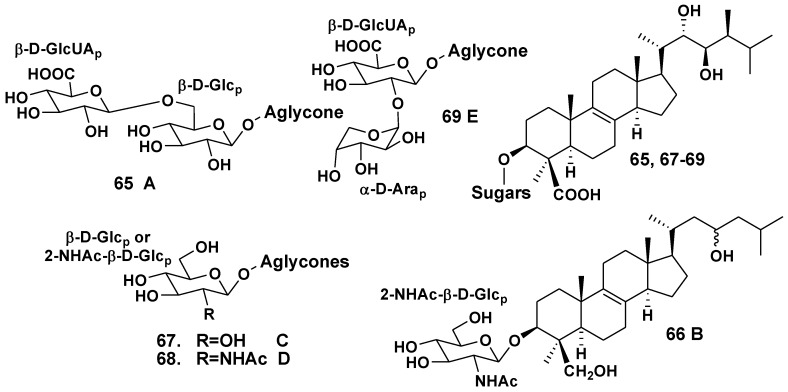
Ulososides from *Ulosa* sp.

The Indian Ocean sponge *Ulosa* sp. belongs to the family Mycalidae. Antonov *et al*. have isolated five glycosides with oxidized 14-nor-methyl-lanostane type aglycones, namely ulososides A–E (**65**–**69**) from the sponge collected off the waters of the North-Western Madagascar [37,38,39]. Ulososides A (**65**) [37], C (**67**), D (**68**) and E (**69**) [39] possess 22*S*,23*R*-diol fragment and 24*S*-methyl in side chains of their aglycones, like brassinolides. Ulososide B (**66**) contains a 23ξ-hydroxy group in its aglycone side chain, but has no 24-methyl group [38]. Compounds **65 **is a bioside with a rare 1,6-bond between D-glucose and terminal D-glucuronic acid, while **66**–**68** are monosides, containing 2-(*N*-acetylamino)-2-deoxy-β-D-glucose or D-glucose as carbohydrate moieties. Like **65**, ulososide E (**69**) is a bioside, but its carbohydrate chain has another monosaccharide composition with glucuronic and uncommon α-D-arabinopyranose as a terminal sugar (Chart 8).

**Chart 9:**
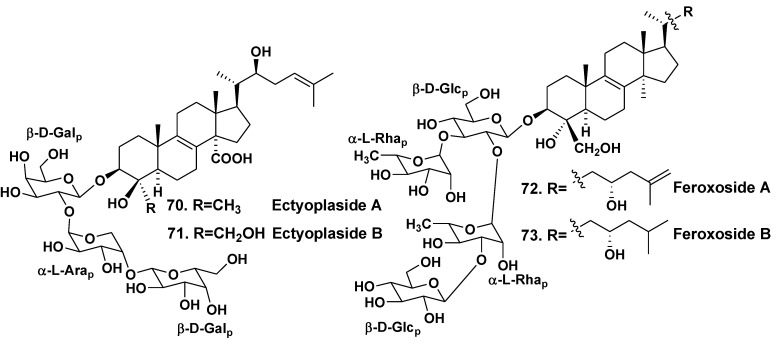
Feroxosides from *Ectyoplasia ferox*.

*Ectyoplasia ferox* is a Carribean sponge belonging to the family Raspailliidae. Four 4β-nor-lanostane triterpenoid glycosides (**70**–**73**), containing oxidized side chains and in the ring A penasterol derivatives as aglycones have been indicated in the glycoside fraction from the sponge collected off the Bahamas [40,41]. Glycosides **70** and **71** are linear triosides, containing two β-D-galactopyranose and α-L-arabinopyranose units. Feroxosides **72** and **73** are tetraosides branched at the first monosaccharide residue with two β-D-glucopyranoses and two α-L-rhamnopyranose residues. α-L-Rhamnose units were never found in sponge triterpene tetracyclic glycosides before the study (Chart 9). Glycosides **70** and **71** showed moderate cytotoxic activities *in vitro* against J774 (murine monocyte-macrophage), WEHI164 (murine fibrosarcoma), and P388 (murine leukemia) cell lines at IC_50_ ranging from 8.5 to 11.4 μg/mL [40]. Glycosides **72** and **73** were also moderately cytotoxic (IC_50_ of 19 μg/mL) against the murine monocyte-macrophage cell line [41].

## 3. Other Triterpene Glycosides from Sponges

### 3.1. Orders Astrophorida and Halichondrida

**Chart 10:**
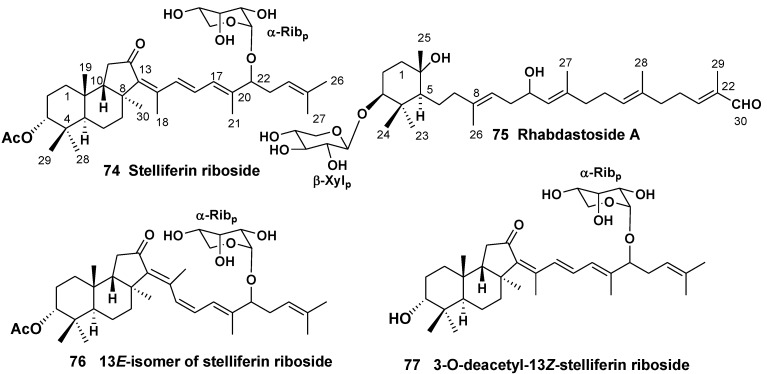
Isomalabarican triterpene glycosides from *Geodia* sp. and *Rhabdastrella globostellata*.

Isomalabaricane triterpene pigments, containing 4,4,8,10-tetramethyl-perhydrobenz[*e*]indane core with a *trans-syn-trans* ring junction were first reported from the Fijian collection of the sponge *Jaspis stellifera* (the family Ancorinidae) [42] and the Somalian sponge *Stelletta* sp. (the family Ancorinidae) [43]. Later they were found in several other sponges, belonging to the order Astrophorida. Some free triterpenoids of this structural group demonstrate potent antiproliferative properties and promotion of DNA polymerase β binding to DNA, but their glycosylated forms were rarely isolated. The first glycosylated isomalabarican triterpenoid, stelliferin riboside (**74**) with cytotoxic properties was found in unidentified species of the genus *Geodia* collected off Fiji Islands [44]. Recently, a new monocyclic triterpene xylopyranoside, rhabdastoside A (**75**), containing a skeleton related to isomalabaricanes system, was isolated along with several new isomalabaricane derivatives from the methanol extract of the sponge *Rhabdastrella globostellata* (the family Ancorinidae) (Chart 10) [45]. Compound **74 **stabilizes DNA polymerase β binding to DNA inducing 29% binding at the concentration 28 μg/mL [46]. Two additional ribopyranosides, 13*E*-isomer of stelliferin riboside (**76**) and 3-*O*-deacetyl-13*Z*-stelliferin riboside (**77**), belonging to this series were isolated from another collection of *R. globostellata*. For their extremely high and selective activities against the mouse lymphoma L5178Y cancer cell line, the compound **76** was found to be more active than **77** with ED_50_ values of 0.22 and 2.4 nM, respectively [47].

The sponge *Asteropus* sp. (the family Ancorinidae) was used by Schmitz’s group to isolate a series of triterpene galactosides, pouosides A–E (**78**–**82**) from the animals collected in Pou Bay of Truk Lagoon in 1979 [48]. It is of special interest that simultaneously they isolated sarasinosides from the same sponge [13]. Pouosides contain a new carbon skeleton of aglycones, arised from cyclization of squalene. This skeleton system is reminiscent of carotenoids of terminal cyclohexane rings linked by a symmetric acyclic chain. A cytotoxicity of pouoside A (**78**) was established [48].

**Chart 11:**
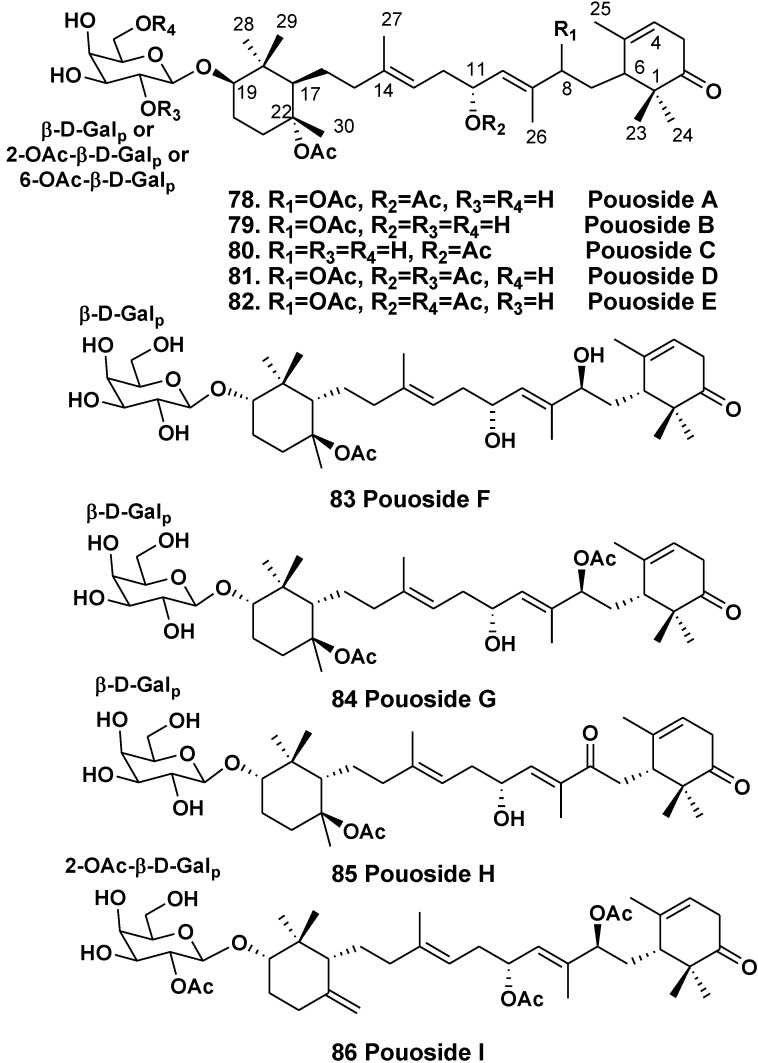
Pouosides from *Lipastrotethya* sp.

Recently, a Korean group has found the same class of compounds in the sponge *Lipastrotethya* sp. (the order Halichondrida, the family Dictyonellidae) collected in Micronesia along with previously known sarasinosides. In total, they isolated nine triterpenoids, pouosides F–I (**83**–**86**) belonging to the same structural series (Chart 11) [49]. It was the unique report about the isolation of triterpene glycosides from a sponge, belonging to the order Halichondrida.

Absolute configurations of **83**–**86** were established by Mosher’s method. Formulae of all the pousides were corrected in accordance with these data. Some pouosides, namely pouoside A and pouosides F–I, demonstrated strong or moderate cytotoxicities against the P-388 and K562 cell lines [48,49].

### 3.2. The Order Haplosclerida

The sponge *Siphonochalina* (=*Callispongia*)*siphonella* (the family Callispongiidae) inhabits different areas in the Red Sea. Squalene-derived triterpenes, having a new sipholane skeleton system (**87**) were first isolated from *S. siphonella* by Kashman *et al*. in 1981 and their structures were established using X-ray analysis [50]. About 20 compounds, having not only sipholane, but also close related siphonellane and neviotane skeleton systems were later reported [51,52]. These natural products and their semisynthetic analogs were evaluated for cytotoxicity and effect on reversing P-glycoprotein-mediated multidrug resistance to colchicin. Some of them increased the sensitivity of resistant KB-C_2_ cells to the cytotoxin [53]. In majority, triterpenoids of this series present in different collections of the sponge as non-glycosylated derivatives. However, several triterpene glycosides were also found. For example, sipholenosides A and B (**88**,**89**) were isolated from *S. siphonochalina*, collected from the Gulf of Eilat *vs.* Dahlak archipelago, Red Sea. The both glycosides are α-rhamnopyranosides [54]. The originally proposed absolute configuration of sipholenol A, a main triterpene of this series was corrected by the high-field NMR application of Mosher’s method [55], herein we give the corrected structures of **88**, **89**.

**Chart 12:**
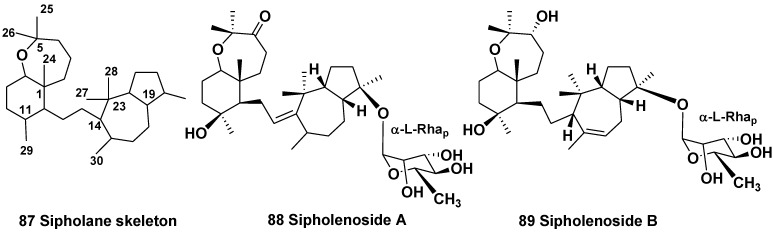
Sipholenosides from *Siphonochalina* (=*Callispongia*) *siphonella*.

Sipholane derivatives belong to an interesting group of triterpenoids, which attract attention not only by their structural diversity (Chart 12), but also by strong biological activities [52].

**Scheme 1:**
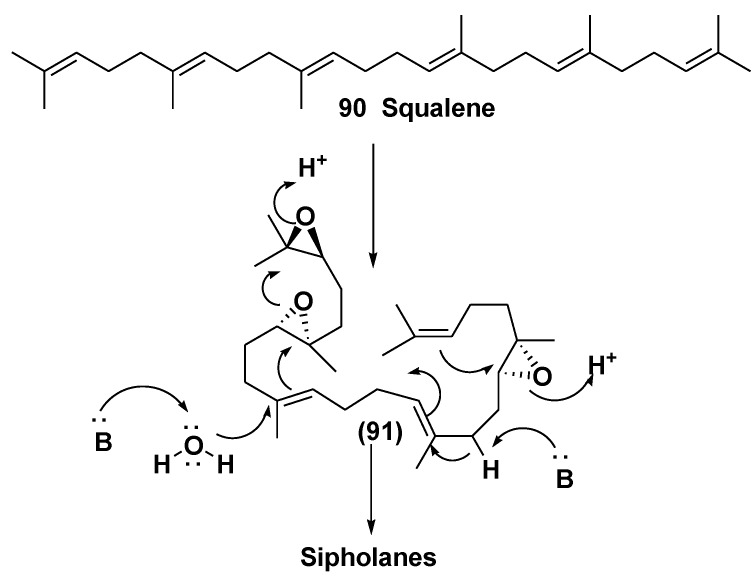
Biogenesis of sipholane triterpenoids.

Sipholanes comprise two separate cyclic systems, obtained by two proton-initiated cyclizations from triepoxysqualenes. The precursor, suggested for their biosynthesis is (2,3*S*,6*S*,7*S*,18*S*,19*S*)-triepoxysqualene (**91**) (Scheme 1).

**Chart 13:**
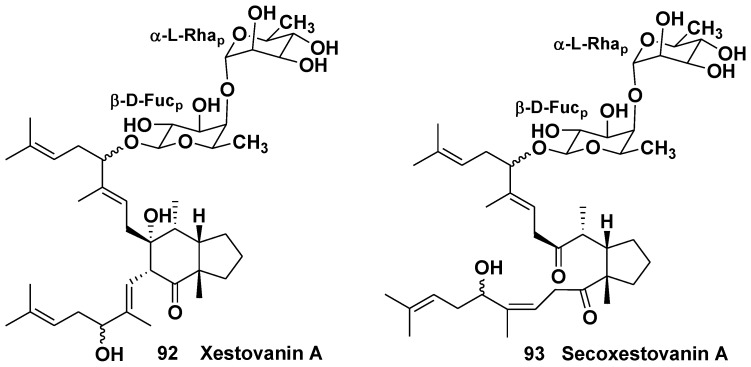
Xestovanin A and secoxestovanin A from *Xestospongia* (=*Neopetrosia*) *vanilla*.

**Chart 14:**
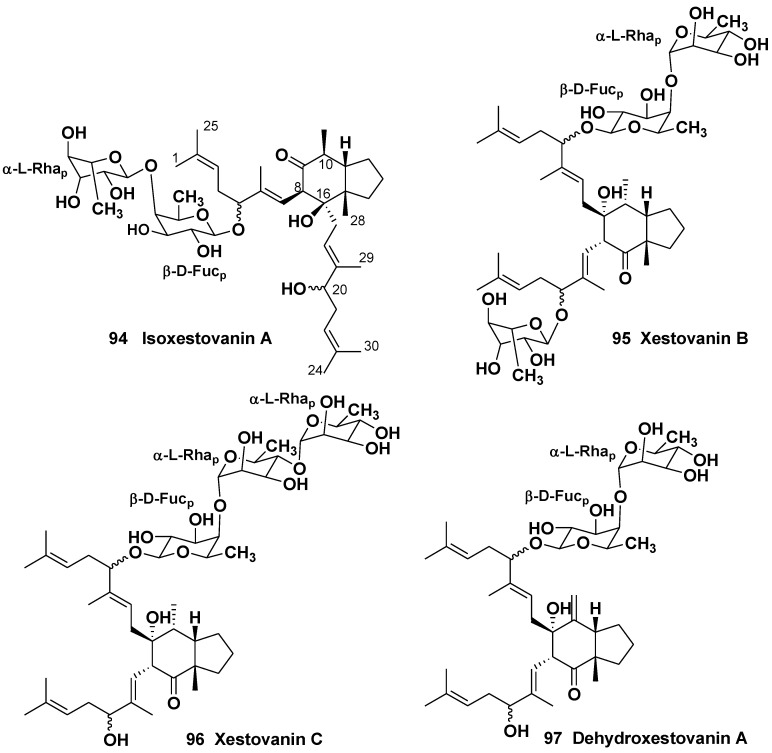
Isoxestovanin A, xestovanins B and C and dehydroxestovanin A from *Xestospongia* (=*Neopetrosia*) *vanilla*.

**Chart 15:**
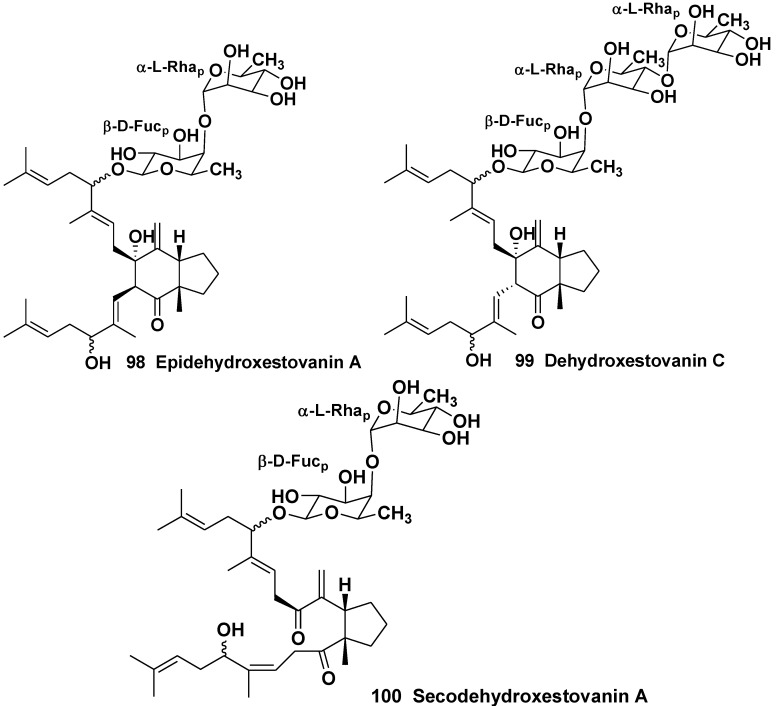
Epidehydroxestovanin A, dehydroxestovanin C and secodehydroxestovanin A from *Xestospongia* (=*Neopetrosia*) *vanilla*.

The sponge *Xestospongia* (=*Neopetrosia*) *vanilla* (the suborder Petrosina, the family Petrosiidae) inhabits underwater areas near the Californian coast-line (Canada and USA). Two first triterpene glycosides, xestovanin A and secoxestovanin A (**92**, **93**) with new skeleton systems were found in *X. vanilla* by Northcote and Andersen in 1989 [56]. Aglycone moiety in **92** is formed by unusual 11,15- and 9,17-squalene cyclizations. Both xestovanin A and secoxestovanin A belong to triterpene biosides and contain carbohydrate chains with 1,2-bonded D-fucose and L-rhamnose (Chart 13). Xestovanin A (**92**) shows antifungal activity against *Phytium ultimum*. Later, the same group of scientists reported the isolation and structure elucidation of 7 new related glycosides (**94**–**100**) (Chart 14 and Chart 15). The aglycone of isoxestovanin A (**94**) has a new triterpene carbon skeleton, named isoxestovanine. Xestovanins B, C and dehydroxestovanin C (**95**, **96**, **99**) are triosides, containing two L-rhamnose and D-fucose residues in their carbohydrate moieties [57].

## 4. Steroid Glycosides from Sponges

Sponge steroid glycosides are becoming a growing group of natural products at the last years. Many of them are interesting in both their structural diversity and biological properties.

### 4.1. The order Astrophorida

A steroidal bioside pachastrelloside A (**101**) from the sponge *Pachastrella* sp. (the family Pachastrellidae) induces the formation of multinucleated, unicellular embryos at the action on fertilized eggs of sea urchins. Pachastrelloside A has the sterol aglycone, oxidized in the rings A and B, with two monosaccharide residues (D-galactopyranosyl and 4-*O*-acetyl-β-D-xylopyranosyl) linked to C-4 and C-8 of its aglycone [58].

**Chart 16:**
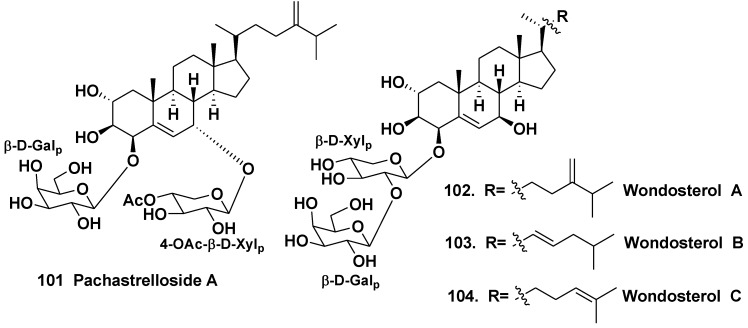
Pachastrelloside A and wondosterols A–C from a two sponge association.

A Korean two-sponge symbiotic association was also studied. This association was composed of *Poecillastra wondoensis* (the family Vulcanellidae) and *Jaspis wondoensis* (the family Jaspidae): the upper part is *P. wondoensis* and the underpart is *J. wondoensis*. The sponge was collected at a depth of 15 m off Cheju Island, South Korea. Three steroidal glycosides, wondosterols A–C (**102**–**104**) were isolated and their stereostructures were elucidated on the basis of NMR spectroscopic analysis with application of Mosher’s method. Structures of **102**–**104** proved to be closely related to that of **101**, although **102**–**104 **contain only one carbohydrate chain linked to C-4 in contrast with **101** (Chart 16).Their carbohydrate moieties consist of D-galactose and D-xylose [59].

The Caribbean sponge *Pandaros acanthifolium* from the family Microcionidae was collected at the Canyon rock off the Martinique. The studies on the chemical composition of the sponge led to the isolation of a series of new steroid glycosides, named pandarosides. Pandarosides A–D (**105**–**108**) were structurally identified as glycosides, containing unusual sterol aglycones with a rare cis-C/D ring junction, oxidized in ring D and side chains.

**Chart 17:**
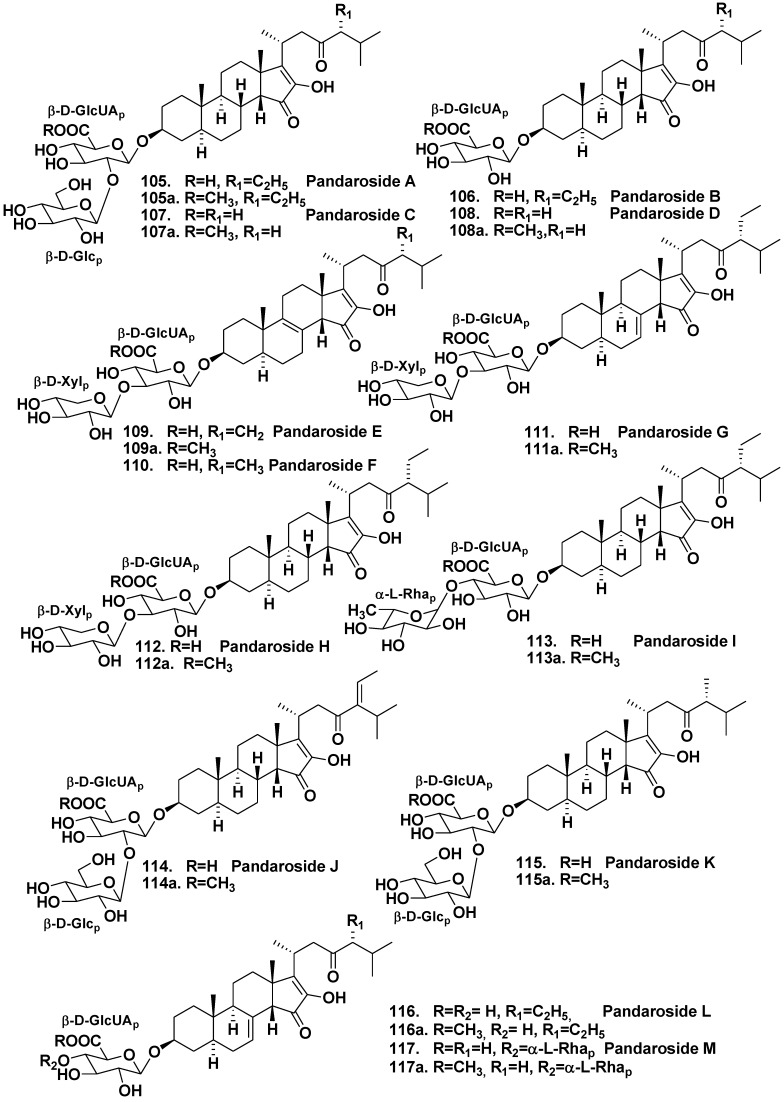
Pandarosides from *Pandaros acanthifolium*.

Their carbohydrate moieties consist of D-glucose and D-glucuronic acids or D-glucuronic acid only at C-3 of the aglycone (Chart 17).

Methyl esters (**105a**, **107a** and **108a**) of pandarosides were also isolated. Absolute configuration of an aglycone part of **105** was established by comparison between calculated and experimental circular dichroism spectra [60]. After the isolation of additional steroidal glycosides, pandarosides E–J (**109**–**114**) and their methyl esters (**109a**, **111a**–**114a**), it was shown that the majority of pandarosides exhibit *in vitro* activity against three of four tested parasitic protozoa. Pandaroside G (**111**) and its methyl ester (**111a**) are potent inhibitors of the growth of *Trypanosoma brucei rhodesiense* (IC_50_ of 0.78 and 0.038 μM, respectively) and *Leishmania donovani* (IC_50_ of 1.3 and 0.051 μM, respectively) [61]. Finally, pandarosides K–M (**115**–**117**) and methyl esters (**115a**–**116a**) were also isolated as minor components after careful chemical reinvestigation of the same species [62]. However, pandarosides K–M did not show strong antiprotozoal activities in contrast with pandaroside G.

**Chart 18:**
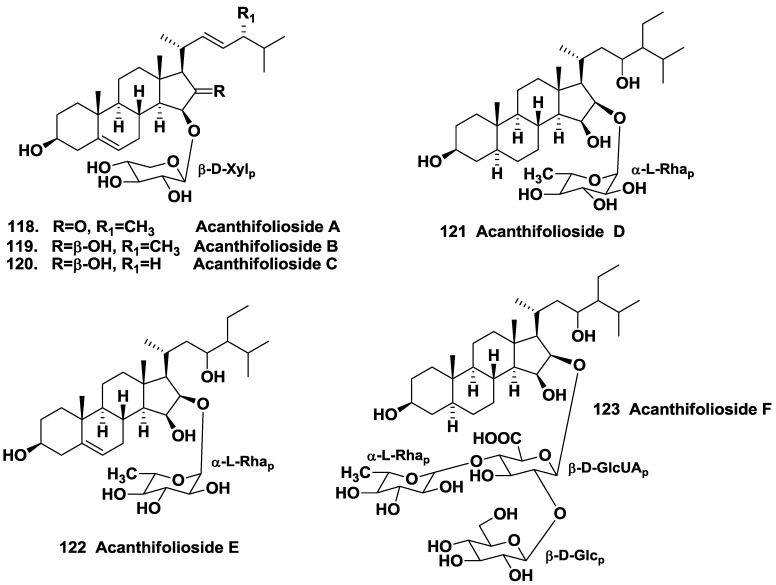
Acanthifoliosides from *Pandaros acanthifolium*.

Other steroid glycosides from the same sponge were named as acanthifoliosides A–F (**118**–**123**). Acanthifoliosides are structurally related to pandarosides steroid saponins, but they contain common steroid nuclei with *trans*-junction of rings C and D. Like aglycones of pandarosides, aglycones of **118**–**123** were oxidized in the ring D. Carbohydrate chains of the glycosides is linked to C-15 (in compounds **118**–**120**) or to C-16 of aglycone moieties (in compounds **121**–**123**). Acanthifoliosides A–C (**118**–**120**) were proved to be mono-β-D-xylopyranosides, acanthifoliosides D and E (**121**–**122**) are mono-α-L-rhamnopyranosides, while acanthifolioside F (**123**) contains a branched trioside carbohydrate chain (Chart 18). Acanthiofoliosides exhibit moderate antiprotozoal activities [63].

There is an evident structural similarity between glycosides from *P. acanthifolium* and steroid glycosides from starfish, because oxidation in positions 15, 16 and 23 is frequently encountered in both these groups of natural products [7,8,9].

For the first time, steroid oligoglycosides similar to asterosaponins by containing several sugar units were found in a sponge, belonging to the genera *Mycale* (the family Mycalidae) by our group in 1981 [64]. However, successful separation of very complicated glycoside fractions from the sponge was not achieved in that time. Twenty years later, steroid oligoglycoside mycaloside A (**124**) was obtained from the Caribbean sponge *Mycale laxissima* as individual compound [65].

**Chart 19:**
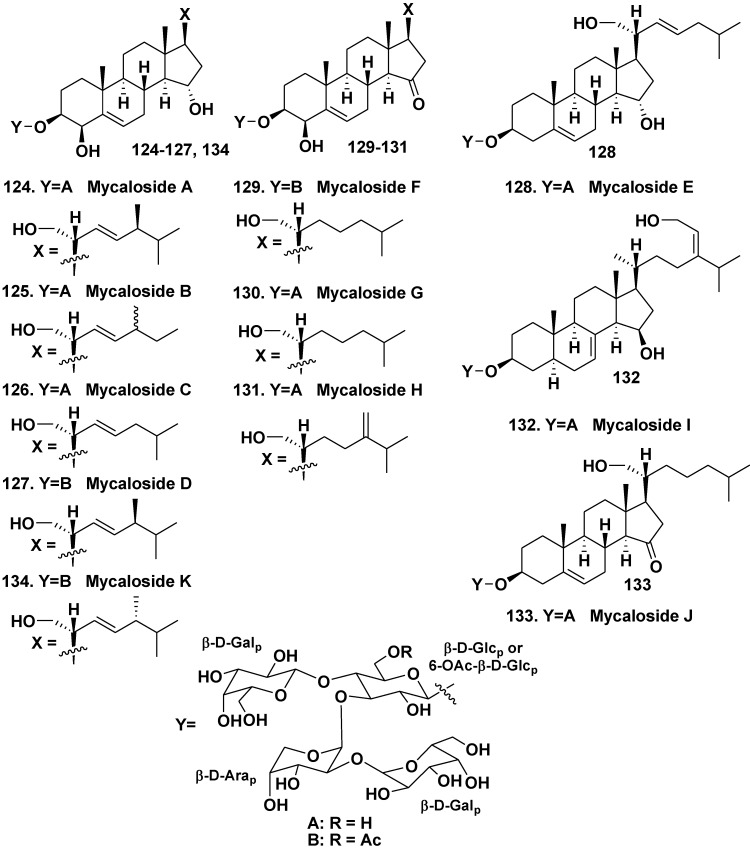
Mycalosides from *Mycale laxissima*.

In subsequent papers, structures and isolation of related mycalosides B–K (**125**–**134**) were reported [66,67]. All the glycosides are tetraosides containing the same architecture of carbohydrate chains, consisting of two D-galactopyranose, one D-glucopyranose, and one D-arabinopyranose residues. Their steroid aglycones are sterol derivatives, oxidized in rings A, D, and in side chains. Mycalosides B (**125**) and C (**126**) were shown to be 27- and 28-nor derivatives of (**124**), respectively. Mycaloside D (**127**) differs from **124** only in the presence of an additional acetyl group in the carbohydrate moiety. Mycaloside E (**128**) was structurally identified as a 28-nor-4-deoxy derivative of **124**. Mycalosides F–H (**129**–**131**), differing from each other in structures of their side chains and nonacetylated (**130**, **131**) or acetylated (**129**) tetrasaccharide carbohydrate moieties, have new 5(6)-unsaturated 3β,4β,21-trihydroxy-15-keto-steroidal aglycons. Mycaloside I (**132**) is a tetraoside of a new 7,24(28)-diunsaturated 3β,15β,29-trihydroxystigmastane aglycon. Mycaloside J (**133**) contains a new aglycone, differing from that of **126** in the absence of hydroxy group at C-4. Mycaloside K (**134**) is an epimer of **127** at C-24 (Chart 19). The total fraction of the mycalosides as well as purified mycalosides A (**124**) and G (**130**) inhibit the fertilization of eggs by sperm of the sea urchin *Strongylocentrotus nudus* preincubated with these compounds [66].

The sponge *Cribrochalina olemda* belongs to the family Niphatidae and inhabits tropical waters of Indo-Pacific area. Hapaioside (**135**) from the sponge, collected from a depth of 40 m, Micronesia, contains an unusual aglycone that resembles some steroid hormones by its polycyclic nucleus and has 4-hydroxy-6-oxo-19-norpregnane skeleton. Its sugar was identified as 6-deoxy-β-L-altropyranose-4-acetate [68]. The sugar was earlier reported as constituent of a lipopolysaccharide isolated from mammalian intestinal microorganisms.

### 4.2. The Order Halichondrida

**Chart 20:**
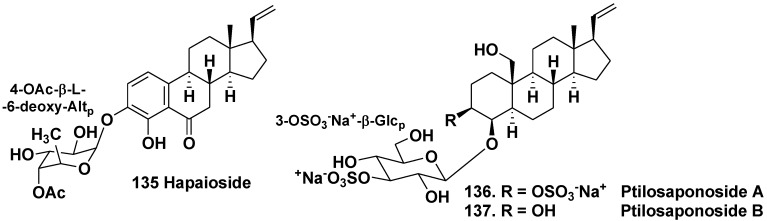
Hapaioside andptilosaponosides from *Ptilocaulis spiculifer*.

The sponge *Ptilocaulis spiculifer* belongs to the family Axinellidae and inhabits the same area as *C. olemda*. *Ptilocaulis spiculifer* contains sulfated polyhydroxysteroids as characteristic secondary metabolites. Pregnane glycosides, ptilosaponosides A (**136**) and B (**137**), along with above-mentioned sulfated natural products were isolated from the sponge collected near the Solomon Islands (Chart 20). They contain β-glucose-3-sulfate as a carbohydrate moiety and have highly oxidized the ring A. These substances also have additional sulfate in the aglycones. Glycosides **136** and **137** do not show a cytotoxicity against human tumor KB cells [69]. Ptilosaponosides are exclusive steroid glycosides found in the order Halichondriidae, but not Astrophorida or Poecilosclerida.

Thus, steroid glycosides from sponges are an interesting group of natural products. Some of them demonstrate a promising antiprotozoal action and other biological activities.

## 5. Glycosides of Non-Isoprenoid Aglycones

Unusual glycosides with non-isoprenoid aglycones, which do not belong to classical glyceroglycolipids and sphingolipids were isolated from extracts of some sponges.

### 5.1. “Atypical” Glycolipids

#### 5.1.1. The Order Astrophorida

**Chart 21:**
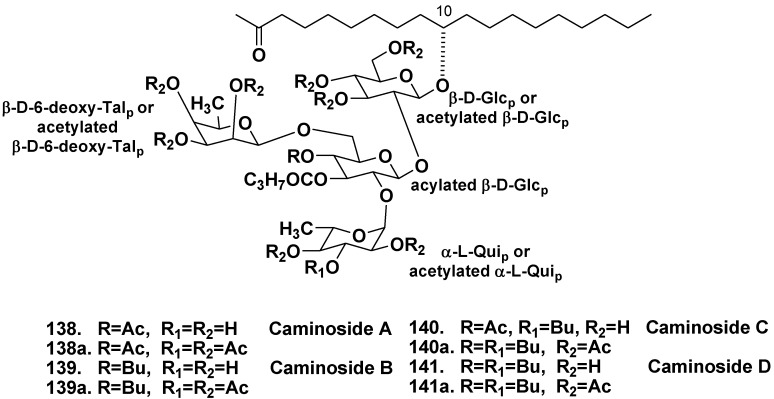
Caminosides from *Caminus sphaeroconia*.

The Caribbean sponge *Caminus sphaeroconia*, from the family Geodiidae, was collected from the depth of 10 m, Toucari Caves, Dominica. As result of a screening program aimed at the search for inhibitors of the bacterial type III secretion, new unusual glycolipids, caminosides A–D (**138**–**141**) were found in extracts of *C. spaeroconia* [70,71]. Caminosides form inseparable mixtures of bioactive glycolipids that have the same tetrasaccharide chain, but differ from each other in acylation level. After acetylation of obtained subfractions of the glycolipids, pure peracetates of caminosides A, C, and D (**138a**, **140a**, **141a**) were isolated using HPLC (Chart 21). For caminoside B (**139**), separation of a crude subfraction by reverse phase HPLC was sufficient to obtain a pure compound without derivatization. Their carbohydrate chains consist of two D-glucopyranose, one L-quinovopyranose and one 6-deoxy-D-talopyranose carbohydrate units. Middle glucose residue is fully substituted. 6-Deoxytalose and L-quinovose are rare in nature. Monosaccharide units are partly acylated. Aglycone is nonadecane derivative oxidized at C-2 and C-10. Stereogenic center at C-10 has recently been shown to have the *R* configuration by a new approach to stereochemical assignment in acyclic lipids named as liposomal exciton-coupling circular dichroism [72]. It is known, that enteropathogenic strains of *Escherichia coli*, responsible for thousands of infant deaths a year, have a specific type III secretion system providing excretion of proteins involved in the construction of a translocation tube between the cytoplasm of the bacterium and the membrane of the host cells. Caminosides A (**138**) and D (**141**) inhibit the excretion and show antimicrobial activities *in vitro*. For example, the compound **138** inhibited the methicillin resistant *Staphylococcus aureus* and vancomycin resistant *Enterococcus* with MIC of 12 μg/mL in the both cases [70]. Later, total syntheses of **138** and **139** were reported [73,74].

**Chart 22:**
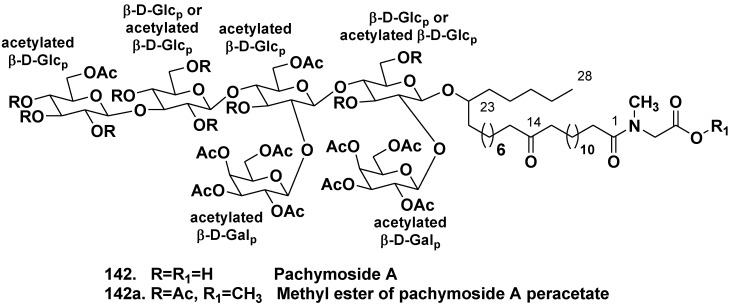
Pachymoside A from *Pachymatisma johnstonia* and its methyl ether.

The sponge *Pachymatisma johnstonia* belongs to the same family Geodiidae as *Caminus spaeroconia*. Crude extracts of the North Sea collection of *P. johnstonia* also showed a promising activity in an assay for inhibitors of bacterial type III secretion. Glycolipids, responsible for the activity were named as pachymosides. Pachymoside A (**142**), a main glycolipid constituent, contains a hydroxylated C_28_ fatty acid derivative as aglycone and hexasaccharide carbohydrate chain, consisting of D-glucopyranose and D-galactopyranose residues with eight acetate groups. All of the metabolites with these variations in acetylation pattern were converted into the same peracetyl-pachymoside methyl ester (**142a**) for purification and spectroscopic analysis (Chart 22) [75].

#### 5.1.2. The Order Poecilosclerida

**Chart 23:**
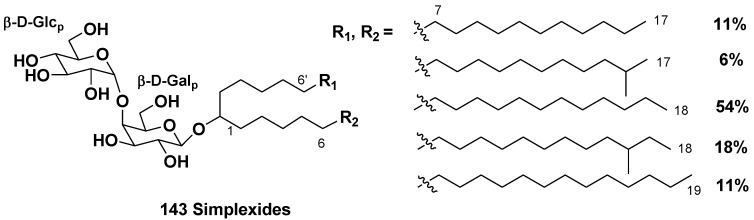
Simplexides from *Plakortis simplex*.

New “atypical” glycolipids, simplexides of general formula (**143)** were isolated from the marine sponge *Plakortis simplex* (the family Plakiniidae) and their structures were determined by spectroscopic data with application of microgram-scale chemical degradation. Simplexides are composed of long-chain secondary alcohols and a disaccharide chain consisting of glucose and galactose, and represent a new structural kind of glycolipids. Simplexides strongly inhibit proliferation of activated T-cells by a non-cytotoxic mechanism. When tested on murine immune-system T-cells stimulated with concanavalin-A, they caused a 43% proliferation inhibition at a concentration as low as 10 ng/mL and 79% inhibition at 100 ng/mL. It was suggested that these compounds can be regarded as simple model molecules for designing immunosuppressive drugs (Chart 23) [76].

#### 5.1.3. The Order Agelasida

**Chart 24:**
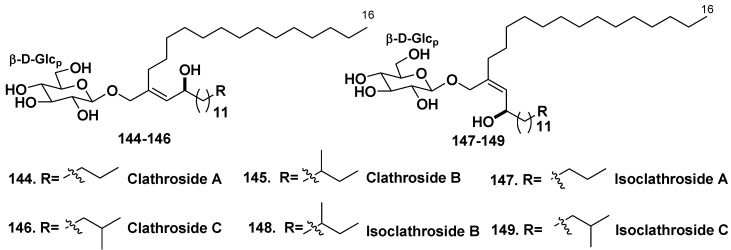
Clathrosides and isoclathrosides from *Agelas clathroides*.

Two families of unique glycolipids, clathrosides A–C (**144**–**146**) and isoclathrosides A–C (**147**–**149**) were isolated from the Caribbean Sponge *Agelas clathroides* (the family Agelasidae, Grand Bahamas Island). Clathroides and isoclathrosides were proved to be glycosides of a very-long-chain alcohol derived from fatty acids (Chart 24). The six compounds differ from each other in configuration and in the branching of alkyl chains. Location of the methyl branch on the proper alkyl chain was difficult and required an exceptional 1D TOCSY experiment, in which coherence was transferred through as many as 13 vicinal couplings. Actions of clathroside A (**144**) and isoclathroside A (**147**) on immune system were assayed, but the compounds did not show any significant activity [77].

### 5.2. Glycosides of Macrolides

#### 5.2.1. The Order Astrophorida

**Chart 25:**
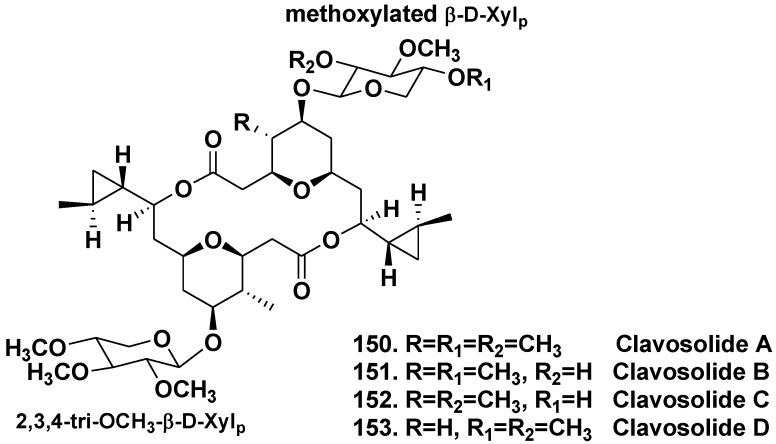
Clavosolides from *Myriastra clavosa*.

The sponge *Myriastra clavosa* from the Philippines belongs to the family Ancornidae and contains symmetric and asymmetric dimeric macrolides, clavosolides A and B (**150**, **151**). The both glycosides are bis-methoxylated-β-D-xylopyranosides, not related to any known sponge metabolites. Taking into consideration the presence of a high concentration of cyanobacterial cells in the sponge, it was suggested that clavosolides might be of cyanobacterial origin [78]. Simultaneous study led to isolation of closely related clavosolides C and D (**152**, **153**), dimeric macrolides incorporating cyclopropyl, tetrahydropyranyl, and glycosidic ring systems, but having a difference in one of monosaccharide moieties (Chart 25) [79].

#### 5.2.2. The Order Poecilosclerida

**Chart 26:**
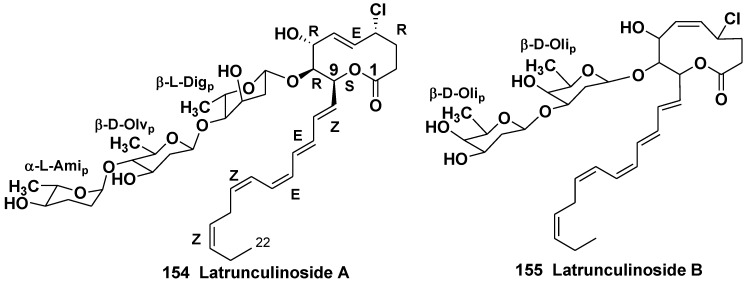
Latranculinosides from *Latrunculia corticata*.

Two novel chlorinated glycosides, latrunculinosides A and B (**154**, **155**) from the sponge *Latrunculia corticata* (the family Latrunculidae) collected off the Gulf of Aqaba, Israel, contain the substituted 2-oxecanones as aglycones and unusual monosaccharides β-D-olivose, β-L-digitoxose, α-L-amicetose, and β-D-oliose (Chart 26). Latrunculinosides were active as antifeedants at concentrations of 10–100 μg/mL in aquarium assays with goldfish (*Carassius auratus*) [80]. This was the first case of finding the glycosides with sugars characteristic of cardiac glycosides in sponges.

### 5.3. Bipolar Glycolipids

#### 5.3.1. The Order Haplosclerida

Containing a β-glycoside moiety two-headed (or bipolar) glycolipids from sponges represent a group of unprecedented *bis*-α,ω-amino alcohol derivatives. Polar heads in these compounds resemble that of sphingoid bases, but one or the both terminal oxymethyl groups in them are replaced by methyl group.

The first representative of this unprecedented group, rhizochalin (**156**) was isolated by our group from the marine sponges *Rhizochalina* (=*Oceanapia*) *incrustata* collected off Madagascar in 1989 [81]. *Oceanapia* spp. (=*Rhizochalina* spp.) belongs to the family Phloeodictyidae. Later, a close related rhizochalin A (**157**) with rare *N*-alkyl carbamoyl group was found in the same sponge [82]. Rhizochalin B (**158**) with an additional butyloxy group was isolated from the close related sponge *Oceanapia* sp., collected off the North-Western Australian coast [83]. Further studies on *R. incrustata* led to the isolation of rhizochalin C (**159**) with a polar head, containing oxymethyl group instead of methyls, along with rhizochalin D (**160**), containing an odd-numbered C_29_ hydrocarbon chain instead of C_28_ that was found in other members of this series [84].

**Chart 27:**
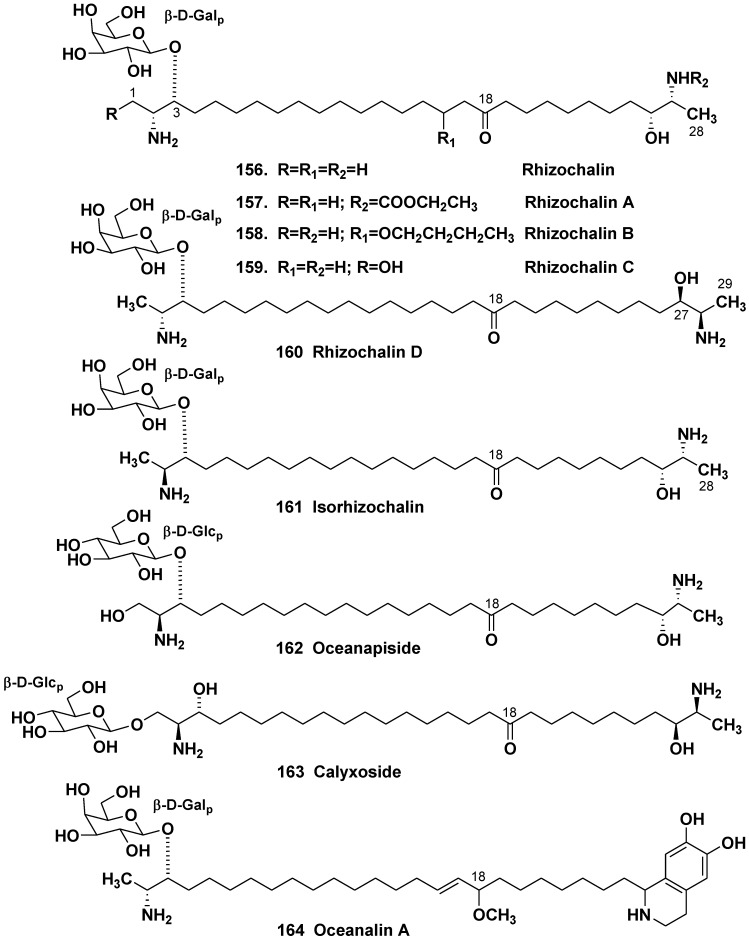
Bipolar glycosidlated lipids from *Oceanapia* (=*Rhizochalina*) spp.

Absolute stereochemistry of asymmetric centers in **156** [85] and related compounds [84] as 2*R*,3*R*,26*R*,27*R* (for **160** as 2*R*,3*R*,27*R*,28*R*) was established by application of a CD method based on superposition of additive exciton couplings in perbenzoyl derivatives of bis-amino alcohols. Absolute stereochemistry at C-2 and C-27 in rhizochalin and related compounds are antipathic in comparison with those in polar heads of sphingolipids, in which *erythro*-configuration takes a place. It was proposed that biosynthesis of terminal polar groups in rhizochalin might be realized via condensation of acyloyl CoA and alanine with loss of CO_2_ and reduction of carbonyl groups on both ends of the molecule. In this case, D-alanine should be employed as a precursor of **156** with retention of configuration at the C-2 and C-27 centers. Isolation of minor isorhizochalin (**161**) [86], an unprecedented epimer of **156** with *erythro* configuration at the glycosylated 2-amino-3-alkanol α-terminus and *threo* configuration on ω-terminus, suggest that α-terminus in this compound may be biosynthesized from L-alanine, while ω-terminus from D-alanine. D-Alanine may be originated from a host-derived epimerase or a symbiotic bacterium in the sponge (Chart 27) [86].

Another related bipolar glycosylated lipid, oceanapiside (**162**), was isolated from the sponge *Oceanapia phillipensis* [87]. Its absolute stereochemistry was established as a result of elaboration of a general CD method [88] that allowed the simultaneous determination of the local absolute configuration at each of the termini of the long chain bis-aminolipids. Like isorhizochalin, the compound **162** was also suggested to be a product of enantiodivergent biosynthesis [88]. The same method [88] was applied also at structural study on another member of the same series, calyxoside (**163**) from the sponge *Calyx* sp. [89]. Two-headed β-glycoside oceanalin A (**164**), a unique natural compound of hybrid alkaloidal sphingolipid, was also isolated from the marine sponge *Oceanapia* sp. [90]. The remarkable finding associated to oceanalin A structure was the discovery of unprecedented confluence of sphingolipid and isoquinoline pathways in marine natural product biosyntheses.

As result of the studies on biological properties of these bipolar glycosides, antibacterial activity against *Staphylococcus aureus*, cytotoxic activity against mouse Ehrlich carcinoma cells [81], antifungal activity against the pathogenic fluconazole-resistant yeast *Candida glabrata* [87,90], selective DNA-damaging action [89], anticarcinogenic [91], and proapoptotic properties [91,92,93] at their micromolar concentration were indicated.

#### 5.3.2. The Order Astrophorida

**Chart 28:**
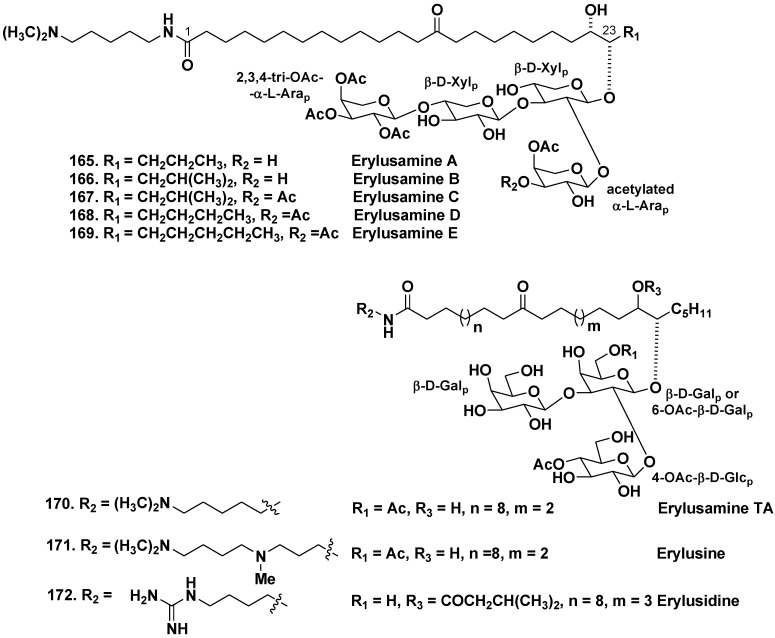
Erylusamins from *Erylus placenta*, erulisine and erulisidine from *Erylus* cf. *lendenfeldi*.

Unprecedented bipolar lipids from the Japanese specimen of the sponge *Erylus placenta* (the family Geodiidae) were named as erylusamines A–E (**165**–**169**). Unusual structural features of these compounds include two polar termini of molecules: one end linked with tetrasaccharide moiety and another one, bearing substituted cadaverine moiety. It was shown that erylusamines are potent IL-6 receptor antagonists (Chart 28) [94,95].

Similar unusual secondary metabolites erylusamine TA (**170**), erylusine (**171**), and erylusidine (**172**) were isolated from the Red Sea sponge *Erylus* cf. *lendenfeldi*. Like erylusamines, these unique bipolar lipids contain asymmetrically located carbonyl in a long hydrocarbon chain, two polar termini and partly acetylated trisaccharide, but not tetrasaccharide as in erylusamines carbohydrate chains [96]. Sugars in all this group of unusual glycosylated bipolar lipids were identified as β-D-galactopyranose, β-D-glucopyranose, α-L-arabinopyranose, their acetylated derivatives, and β-D-xylopyranose.

## 6. Taxonomic Distribution of Glycosides in Sponges

As a rule, triterpene and steroid glycosides present in sponges as very complicated mixtures and their separation is difficult. This may cause some problems in the use of these substances as chemotaxonomic markers, because investigators may sometimes isolate only a part of components from complicated glycoside fraction while another group of investigators may isolate another part of the components. As result, the comparison of these data may be not correct. On the other hand, distinct structural series of glycosides are evidently distributed in different and concrete taxa of the animals. Distribution of tetracyclic triterpene and steroid glycosides is mainly limited to two orders (Astrophorida and Poecilosclerida) with a few exceptions, by several families and species within them without a strict dependence on ecological and geographical factors. For example, taxonomic distribution of tetracyclic triterpene glycosides, given in Table 1, testify the presence of sarasinosides with sarasinoside A_1_ (**1**) as prominent constituent in *Melophlus sarasinorum*, collected in different geographical areas from the North-Western Australia to Guam. Sarasinoside A_3_ (**3**) was isolated from *M. sarasinorum* collected off Palau Islands, Guam Island, Sulawesi Island and the North-Western Coast of Australia, sarasinoside A_2_ (**2**)—near Palau Island and from North-Western Coast of Australia, *etc*. The similar distribution was indicated in relation of erylosides of *Erylus formosus*, from which formoside (**24**) and eryloside F (**25**) were isolated using collections carried out off the Bahamas and Puerto Morelos in the Gulf of Mexico.

As we already have noted, recently, a Korean group reported on the isolation of a series of sarasinosides from the sponge *Lipastrotethya* sp. (the order Halichondrida, the family Dictyonellidae) collected in Micronesia [49]. However they did not report the structures of these substances. This finding is so uncommon that it rather seems that such identification may be a result of an error in collecting and identification of sponges.

Although findings the same glycosides in different sponge species, belonging to the same genus is very rare (now there is only one such finding: the isolation of eryloside H (**33**) from *Erylus formosus* collected near Puerto Morelos and from *E. nobilis* collected off Jeju Island, the South Korea), some genera are characterized by the presence of close related glycosides, as it may be exemplified by erylosides found in different species of *Erylus*, collected off three Oceans (Atlantic, Indian and Pacific). All the glycosides contain 14-carboxy- or 14-nor-methyl-lanostane aglycones, sometimes additionally dealkylated at C-4 and alkylated in side chains. General architectures of carbohydrate chains from a series of glycosides isolated from *Erylus* spp. are frequently similar to each other, but, as a rule, exact structures are specific.

Therefore, triterpene, steroid and other glycosides may be considered as taxonomic markers specific on species and sometimes on genus or subgenus levels. However, practical usage of this character for improvement of sponge taxonomy seems to be rather difficult not only due to the presence of very complicated mixtures of close related glycosides, but also seasonal and ecological variability in content of some glycoside components in glycoside fractions. For instance, Kubanek *et al*. [97] indicated the presence of formoside in *Erylus formosus* collected off the Bahamas and its absence in the Floridian collection. It is of special interest that sometimes triterpene glycosides, containing non-tetracyclic aglycones are distributed in the same taxa as those with tetracyclic aglycones. Moreover, some of them accompany each other. For example, pouosides (**78**–**82**) were found in *Asteropus* sp. (=*Melophlus* sp.) together with sarasinosides [13,48].

**Table 1:** Taxonomical distribution of tetracyclic triterpene glycosides in sponges of the class Demospongiae.

Taxon			Place of collection	Glycosides	Reference
Order Astrophorida					
	Family Ancorinidae				
**		*Melophlus* sp.	Guam Island, Truk Lagoon	**1**	[13]
**		Melophlus	Palau Islands	**1, 4, 5**	[12]
**		sarasinorum			
–			–	**1–9**	[15]
–			Solomon Islands	**5, 10–13**	[16]
–			Guam Island	**1, 3, 14–17**	[17]
–			Sulawesi Island	**1, 3, 15–21**	[14]
–			Reef Scott, North-Western Coast of Australia	**1–3, 20–23**	[18]
	Family Geodiidae				
**		*Erylus formosus*	Bahamas Islands	**24**	[19]
–			–	**25**	[22]
–			–	**24, 26**	[23]
–			Puerto Morelos, the Caribbean Sea	**25, 27–36**	[24]
–			–	**24, 37–43**	[25]
**		*Erylus lendenfeldi*	Gulf of Eilat, Red Sea	**44**	[27]
–			Red Sea, north of Hurghada	**44–46**	[28]
–			Gulf of Aqaba, Red Sea	**45, 47**	[29]
**		Erylus goffrilleri	Bahama Islands	**48**	[30]
–			Arresife-Seko Reef, Cuba	**49–56**	[31]
**		*Erylus* sp.	South of New Caledonia	**57, 58**	[32]
**		*Erylus placenta*	Hachijo Island, South Japan	**59, 60**	[33]
**		*Erylus nobilis*	Jaeju Island, South Korea	**33, 61–63**	[26]
–			Shikine-jima Island, 200 km south of Tokyo, Japan	**64**	[35]
Order Poecilosclerida					
	Family Mycalidae				
**		*Ulosa* sp.	Madagascar	**65–69**	[37,38,39]
	Family Raspaillidae				
**		*Ectyoplasia ferox*	San Salvador Island, Bahamas	**70, 71**	[40]
–			Grand Bahama Island	**72, 73**	[41]

Such findings of different squalene cyclizations indicate that *de novo* biosynthesis of triterpene systems may take place in sponges.

Glycosides of non-isoprenoid aglycones, including glycosylated macrolides, and nontetracyclic triterpene glycosides were also found in the same orders of Demospongiae as triterpene and steroid glycosides, in majority cases in Astrophorida and Poecilosclerida.

It confirms the presence of specific glycosyltranferases, participating in construction of oligoglycoside chains in a group of close related representatives of these taxa. Moreover, the same species contain sometimes triterpene glycosides and oligoglycosylated bipolar lipids as may be exemplified by *Erylus placenta*, from which sokodosides [33] and erylusamines [94,95] were isolated. However, a few glycosides of these subclasses of glycosylated secondary metabolites were isolated from representatives of Halichondrida. Moreover, glycosides of alicyclic aglycones seem to have wider distributions in comparison with others subclasses, because they were isolated also from some sponges belonging to the Agelasida and Haplosclerida orders. We think that many new glycosides will be isolated from these and other sponge taxa in the near future.

## 7. Biological Roles of Sponge Glycosides

Sponges have always been an important component of coral reef communities [98]. Kubanek *et al*. [97] have found that glycosides from *E. formosus* collected off the Bahamas and the Southern Florida deters predation by the fish *Thalassoma bifasciatum*. Formoside (**24**) and formoside B (**26**), having four sugars in carbohydrate chain, were less potent than the non-separated fraction of hexaosides, containing the same penasterol as an aglycone. The total glycoside fraction was more active than any subfraction obtained at its separation. Thus, glycosides may play a defensive role against predatory fish.

The studies on glycosides from two the Caribbean sponges *Erylus formosus* and *Ectyoplasia ferox*, showed influence of these substances not only on fish feeding, but also on attachment by biofilm-forming bacteria, fouling by invertebrates and algae as well as on overgrowth by neighboring sponges (allelopathy) in field and laboratory assays. The multiple ecological functions of these substances were established [98,99,100,101]. An antifouling role of the glycosides is connected with their antimicrobial properties, reported for sarasinoside J (**18**) [14], some erylosides [27,28,29], sokodosides [33], caminosides [70,71] and other compounds. Strong antiprotozoal activities, for example reported for pandaroside G [61], make also a contribution into protective action against pathogenic microorganisms and fouling. Allelopathic effects of glycosides may be caused by strong cytotoxic activities of many glycoside substances [13,17,27,47], including their influence on embryos of competitive species, as it was reported for sarasinoside A_1_ (**1**) [13], pachastrelloside A (**101**) [58], and rhizochalin [93].

Ecological activities of the glycosides may be affected by small differences in their molecular structures. There was not found a significant excretion of some triterpene glycosides in sea water, but concentration of the glycosides on surface of sponge species was high. Hence, enemies and competitors of these sponges may be deterred by surface contact with triterpene glycosides rather than by contacts through the sea water [97]. However, an important role of sponge triterpene glycosides in chemical signaling, that suggest their distribution in surrounding environment was also recently discovered. The molecular mechanisms, explaining how predators detect deterrent glycosides were established. Zebrafish (*Danio rerio*) rejected artificial diet with the sponge chemical defensive compounds. Transcripts, made from a zebra fish cDNA library were expressed in *Xenopus laevis* oocytes and tested for chemoreceptor activation via electrophysiology. Electrophysiological response to formoside and ectyoplasides A and B was indicated [102]. In continuation of the study, a new RAMP-like triterpene glycoside receptor (RL-TGR), involved in chemical signaling was found in predatory fish. This receptor responds to glycosides as chemical defensive compounds in the marine environment [103].

An influence of sponge glycosides on membrane properties of host cells is an obvious peculiarity of biological functions of some glycosides, in particular, atypical glycolipids, including unusual bipolar compounds. Structures of the latter compounds suggest their inclusion into lipid bilayer with location of polar heads on the both its inner and outer surfaces. Many of this type of compounds demonstrate antimicrobial properties [70,71,81,87,90]. It was shown that alterations in kinase cascades and apoptosis of treated by these compounds cells are induced at action of bipolar lipids from sponges on different cellular models [91,92]. All these effects may be connected with defensive, antifouling and allelopathic functions of sponge glycosides.

## 8. Conclusions

About 170 unusual sponge glycosides were isolated, in majority cases from sponges belonging to species of orders Astrophorida and Poecilosclerida. The presence of some or other glycoside series, especially triterpene and steroid, is a characteristic feature of a few taxonomic groups (up to date they were indicated in about two dozens of species) within the class Demospongiae, but not of the class as a whole. It seems to be probable that modern improvement in isolation and spectral techniques will facilitate the isolation of many new glycoside compounds from above-mentioned and other sponge taxa. The distribution and biological activities of these compounds suggest their parallel origin and evolution as defensive agents in several taxa of the class.

Chemically, these compounds are very diverse and quite different in the structures of the both aglycone and carbohydrate moieties, when compared with other glycosides of marine or terrestrial origin. Different sponge glycosides have aglycones of a great diversity with alicyclic bi, tri- or tetracyclic aglycones. In many cases, they have previously unknown skeleton systems of aglycones. There are not only mono- and biosides, but also oligoglycosides among compounds, isolated and studied. A large set of monosaccharides (more than two dozens) was found, all are in pyranose forms and the majority is of D-series, although L-sugars were also identified. Such rare sugars as L-quinovose, 6-deoxy-β-L-altropyranose and 6-deoxy-D-talose along with many common monosaccharides were found in carbohydrate moieties of sponge glycosides. Frequently, sponge glycosides contain acylated monosaccharide units. Sponge glycosides demonstrate not only deterrent and ichthyotoxic properties, but also antiprotozoal, antifungal, antitumor and other activities, sometimes very potent and probably connected with their defensive, antifouling, and allelopathic biological functions. Such sponge glycosides as glycosylated acyclic derivatives and unusual bipolar lipids are presumably membrane constituents of sponge cells, modulating their membrane properties.
